# Development and validation of a predictive model assessing the risk of sarcopenia in rheumatoid arthritis patients

**DOI:** 10.3389/fimmu.2024.1437980

**Published:** 2024-07-29

**Authors:** Yuan Qu, Lili Zhang, Yuan Liu, Yang Fu, Mengjie Wang, Chuanguo Liu, Xinyu Wang, Yakun Wan, Bing Xu, Qian Zhang, Yancun Li, Ping Jiang

**Affiliations:** ^1^ First College of Clinical Medicine, Shandong University of Traditional Chinese Medicine, Jinan, China; ^2^ Spinal and Spinal Cord Department, Shandong Wendeng Osteopathic Hospital, Weihai, China; ^3^ Experimental Center, Shandong University of Traditional Chinese Medicine, Jinan, China; ^4^ Rehabilitation College, Shandong University of Traditional Chinese Medicine, Jinan, China; ^5^ Department of Rheumatology, Affiliated Hospital of Shandong University of Traditional Chinese Medicine, Jinan, China; ^6^ Science and Technology Department, Affiliated Hospital of Shandong University of Traditional Chinese Medicine, Jinan, China; ^7^ College of Traditional Chinese Medicine, Shandong University of Traditional Chinese Medicine, Jinan, China

**Keywords:** rheumatoid arthritis, sarcopenia, risk prediction, nomogram, diagnosis

## Abstract

**Background:**

Sarcopenia is linked to an unfavorable prognosis in individuals with rheumatoid arthritis (RA). Early identification and treatment of sarcopenia are clinically significant. This study aimed to create and validate a nomogram for predicting sarcopenia risk in RA patients, providing clinicians with a reliable tool for the early identification of high-risk patients.

**Methods:**

Patients with RA diagnosed between August 2022 and January 2024 were included and randomized into training and validation sets in a 7:3 ratio. Least Absolute Shrinkage and Selection Operator (LASSO) regression analysis and multifactorial logistic regression analysis were used to screen the risk variables for RA-associated muscle loss and to create an RA sarcopenia risk score. The predictive performance and clinical utility of the risk model were evaluated by plotting the receiver operating characteristic curve and calculating the area under the curve (AUC), along with the calibration curve and clinical decision curve (DCA).

**Results:**

A total of 480 patients with RA were included in the study (90% female, with the largest number in the 45–59 age group, about 50%). In this study, four variables (body mass index, disease duration, hemoglobin, and grip strength) were included to construct a nomogram for predicting RA sarcopenia. The training and validation set AUCs were 0.915 (95% CI: 0.8795–0.9498) and 0.907 (95% CI: 0.8552–0.9597), respectively, proving that the predictive model was well discriminated. The calibration curve showed that the predicted values of the model were basically in line with the actual values, demonstrating good calibration. The DCA indicated that almost the entire range of patients with RA can benefit from this novel prediction model, suggesting good clinical utility.

**Conclusion:**

This study developed and validated a nomogram prediction model to predict the risk of sarcopenia in RA patients. The model can assist clinicians in enhancing their ability to screen for RA sarcopenia, assess patient prognosis, make early decisions, and improve the quality of life for RA patients.

## 1 Introduction

Rheumatoid arthritis(RA) is an immune-mediated chronic arthritis characterized by persistent joint pain, with a prevalence of approximately 1% (1). The inflammatory state of the body and inadequate physical activity can lead to a reduction in skeletal muscle mass, with sarcopenia being identified as a significant complication of RA in studies (2). Sarcopenia is characterized by the simultaneous loss of skeletal muscle mass and strength (3). Reduced skeletal muscle mass and strength significantly impair physical functioning in RA patients (4, 5). RA patients with sarcopenia have a significantly higher incidence of vertebral osteoporotic fractures compared to those without sarcopenia (6). Long-term clinical observations have confirmed that sarcopenia can result in decreased physical mobility and a notable increase in falls (7). Research has shown that decreased skeletal muscle mass correlates with increased disability and metabolic abnormalities in RA patients, significantly diminishing their quality of life, incapacitating them, and potentially resulting in mortality (8). The inclusion of sarcopenia in the International Classification of Diseases in 2016 has highlighted its importance. Recent studies have revealed that early nutritional and exercise interventions are effective in halting the progression of sarcopenia (9). It is imperative to identify patients with a high susceptibility to RA sarcopenia for effective management and assessment of RA.

However, there are significant barriers to identifying patients at high risk for RA-associated sarcopenia (7). Although diagnostic criteria for sarcopenia exist, they are not fully applicable to the RA population. First, grip strength (GS) testing is an important tool in the testing of skeletal muscle strength, but patients with RA suffer from joint pain, resulting in skewed GS data that may not accurately reflect the patient’s true skeletal muscle strength. Second, dual-energy X-ray and bioelectrical impedance analysis (BIA) are essential instruments used to detect skeletal muscle mass, but they are not widely available, and most primary care organizations do not have these medical facilities. Finally, clinicians at all levels still pay insufficient attention to diagnosing sarcopenia (10). Consequently, clinical evaluation of RA sarcopenia remains challenging, hindering early recognition by healthcare providers and timely treatment initiation. These issues pose barriers to the management of chronic diseases and the enhancement of quality of life in RA.

This study aimed to develop and validate a risk prediction model for sarcopenia in RA patients. To provide a brief and valuable tool for healthcare providers to identify patients with RA sarcopenia in a timely manner during routine examinations, to implement effective interventions, to optimize clinical outcomes, and to improve the quality of survival of RA patients.

## 2 Method

### 2.1 Study design and patients

The study, conducted at the Affiliated Hospital of Shandong University of Traditional Chinese Medicine, adhered to the principles of the Declaration of Helsinki and received approval from our hospital’s Ethics Committee [approval number: (2022) No. (083)-KY]. Informed consent was exempted by the ethics committee because it was a retrospective study. It retrospectively enrolled RA patients diagnosed (11) between August 2022 and January 2024, aged ≥18 years, excluding those with incomplete skeletal muscle data. Patients were randomly allocated to training and validation sets in a 7:3 ratio. Sarcopenia was diagnosed based on criteria established by the Asian Sarcopenia Working Group (12).

### 2.2 Data collection

Clinical data were collected from the electronic medical records, including gender, age, disease duration, GS, height, weight, BIA report card, Percentage Body Fat (PBF), visual analog scale(VAS), anti-cyclic citrullinated peptide antibody (CCP), rheumatoid factor (RF), C-reactive protein (CRP), erythrocyte sedimentation rate (ESR), Disease Activity Score 28 with C-reactive protein(DAS28-CRP), Disease Activity Score 28 with the erythrocyte sedimentation rate(DAS28-ESR), white blood cells (WBC), neutrophil, neutrophils%, lymphocyte, lymphocytes %, red blood cells, hemoglobin, platelets, alanine aminotransferase (ALT), aspartate aminotransferase (AST), total protein, albumin, Urea, creatinine, uric acid, glucose, triglycerides, cholesterol, high-density lipoprotein (HDL-C), low-density lipoprotein (LDL-C), and glomerular filtration rate (GFR).

### 2.3 Statistical analysis

Data were analyzed using SPSS software (version 27.0) and R software (version 4.2.2). The data are presented as median (interquartile range), or number (percentage) as appropriate. Variables were compared between groups with and without sarcopenia using chi-square tests or Fisher’s exact probability method. The Least Absolute Shrinkage and Selection Operator (LASSO) regression analysis was employed in the training set to screen for factors influencing RA sarcopenia, including statistically significant variables in the multifactorial logistic regression analysis, and further identifying independent influences of RA sarcopenia. Subsequently, the nomogram prediction model for these factors was developed, and internal validation was conducted using the Bootstrap resampling method. The Receiver Operating Characteristic Curve (ROC) was plotted in the training and validation sets, and the Area Under the Curve (AUC) was calculated to evaluate the discriminative power of the predictive model. The consistency between the predicted probability and the actual observation probability was determined by calibrating the curve to evaluate the discriminative power of the nomogram. Finally, the predictive performance and clinical utility of the nomogram were evaluated using the clinical decision curve (DCA).

## 3 Result

### 3.1 Patient characteristics

**Figure 1:**
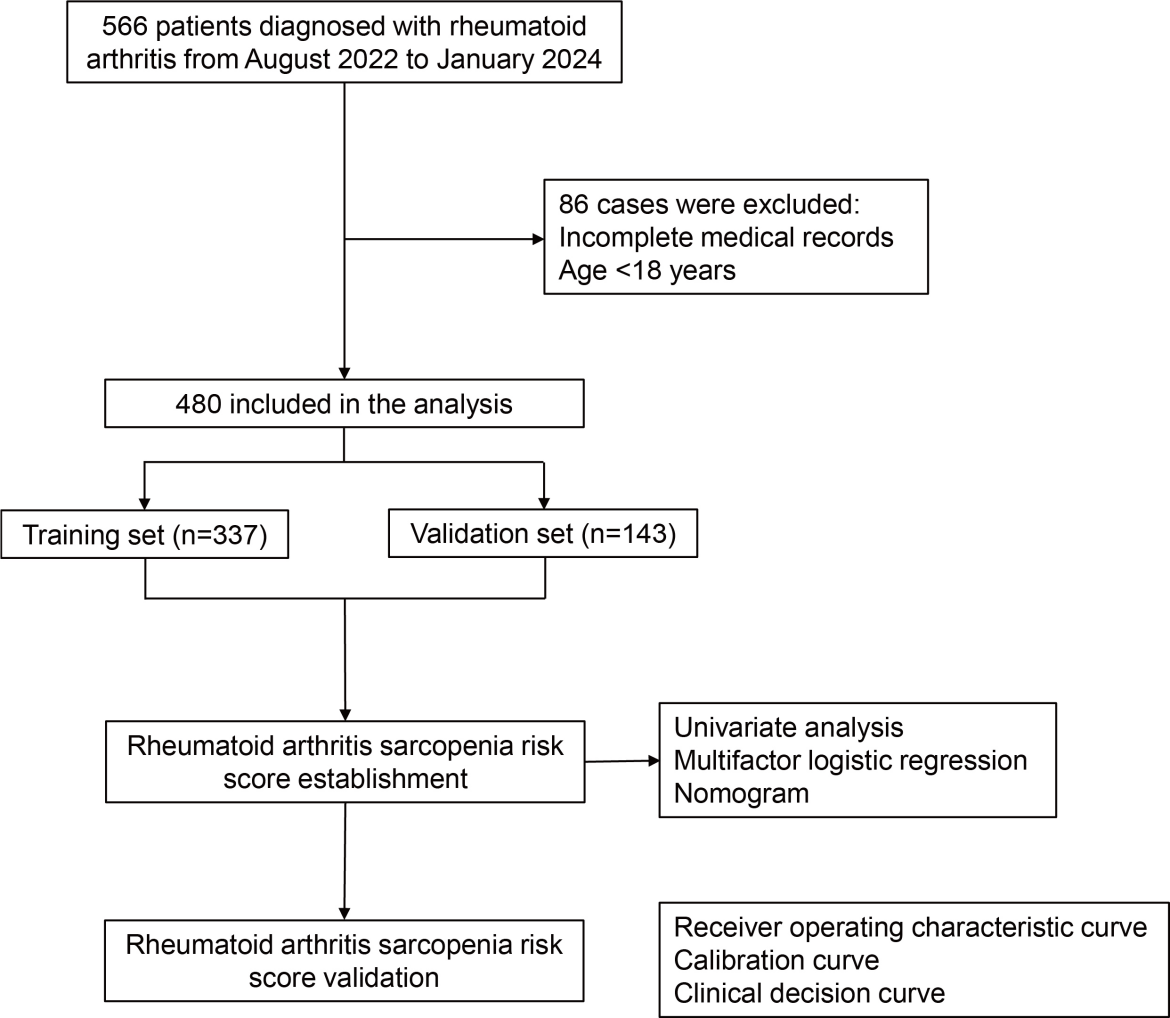
Study profile.

A total of 480 patients with RA were included in the study (**Figure 1**). The training set comprised 271 patients without sarcopenia and 66 patients with sarcopenia. The validation set comprised 116 patients without sarcopenia and 27 patients with sarcopenia. In both cohorts, the male-to-female ratio was approximately 1:9, and the 45–59 age group comprised the largest proportion, approximately 50%. The baseline characteristics of the two study populations are presented in **Table 1**.

**Table 1:** Comparison of clinical data between training and validation sets.

Variables	Total (n = 480)	Training set (n = 337)	Validation set (n = 143)	P-value	χ^2^/Fisher
Diagnosis				0.958	0.003
Non-sarcopenia	387 (81)	271 (80)	116 (81)		
Sarcopenia	93 (19)	66 (20)	27 (19)		
Sex				0.401	0.706
Female	433 (90)	301 (89)	132 (92)		
Male	47 (10)	36 (11)	11 (8)		
Age, years				0.684	0.76
<44	128 (27)	87 (26)	41 (29)		
45–59	251 (52)	176 (52)	75 (52)		
60–74	101 (21)	74 (22)	27 (19)		
Disease duration, years				0.044	8.086
<1	162 (34)	120 (36)	42 (29)		
1–5	196 (41)	124 (37)	72 (50)		
5.1–10	70 (15)	52 (15)	18 (13)		
>10	52 (11)	41 (12)	11 (8)		
GS, kg	19.52 (16.79, 24.19)	19.35 (16.5, 24.15)	19.8 (17.02, 24.4)	0.69	23541
BMI				0.709	Fisher
underweight	16 (3)	13 (4)	3 (2)		
normal weight	186 (39)	127 (38)	59 (41)		
overweight	184 (38)	132 (39)	52 (36)		
obesity	94 (20)	65 (19)	29 (20)		
PBF				0.02	9.803
normal	60 (12)	47 (14)	13 (9)		
mildly obese	155 (32)	112 (33)	43 (30)		
moderately obese	145 (30)	88 (26)	57 (40)		
severe obesity	120 (25)	90 (27)	30 (21)		
VAS				0.617	Fisher
pain-free	158 (33)	106 (31)	52 (36)		
Mild pain	203 (42)	148 (44)	55 (38)		
Moderate pain	108 (22)	76 (23)	32 (22)		
Severe pain	11 (2)	7 (2)	4 (3)		
CCP, RU/ml				0.722	0.651
<25	45 (9)	33 (10)	12 (8)		
25–75	21 (4)	16 (5)	5 (3)		
>75	414 (86)	288 (85)	126 (88)		
RF, IU/ml				0.566	1.137
<15	54 (11)	40 (12)	14 (10)		
15–45	83 (17)	61 (18)	22 (15)		
>45	343 (71)	236 (70)	107 (75)		
CRP, mg/L				0.398	1.845
<3.12	238 (50)	161 (48)	77 (54)		
3.12–10	77 (16)	58 (17)	19 (13)		
>10	165 (34)	118 (35)	47 (33)		
ESR, mm/hr				0.553	1.183
<20	206 (43)	141 (42)	65 (45)		
20–60	216 (45)	157 (47)	59 (41)		
>60	58 (12)	39 (12)	19 (13)		
DAS28-ESR				0.424	2.794
Relief	169 (35)	112 (33)	57 (40)		
Low activity	50 (10)	36 (11)	14 (10)		
Medium Activity	216 (45)	159 (47)	57 (40)		
High activity	45 (9)	30 (9)	15 (10)		
DAS28-CRP				0.209	4.536
Relief	200 (42)	133 (39)	67 (47)		
Low activity	104 (22)	81 (24)	23 (16)		
Medium Activity	156 (32)	108 (32)	48 (34)		
High activity	20 (4)	15 (4)	5 (3)		
White blood cells, 10^9^/L				0.764	0.538
<3.5	25 (5)	16 (5)	9 (6)		
3.5–9.5	433 (90)	305 (91)	128 (90)		
>9.5	22 (5)	16 (5)	6 (4)		
Neutrophil, 10^9^/L				0.549	1.198
<1.8	20 (4)	16 (5)	4 (3)		
1.8–6.3	437 (91)	306 (91)	131 (92)		
>6.3	23 (5)	15 (4)	8 (6)		
Lymphocyte, 10^9^/L				0.862	Fisher
<1.1	62 (13)	42 (12)	20 (14)		
1.1–3.2	406 (85)	286 (85)	120 (84)		
>3.2	12 (2)	9 (3)	3 (2)		
Neutrophil %				0.564	Fisher
<40	8 (2)	6 (2)	2 (1)		
40–70	388 (81)	276 (82)	112 (78)		
>70	84 (18)	55 (16)	29 (20)		
Lymphocyte %				0.282	Fisher
<20	88 (18)	56 (17)	32 (22)		
20–50	386 (80)	276 (82)	110 (77)		
>50	6 (1)	5 (1)	1 (1)		
Red blood cells, 10^12^/L				0.202	Fisher
<3.8	88 (18)	62 (18)	26 (18)		
3.8–5.1	384 (80)	267 (79)	117 (82)		
>5.1	8 (2)	8 (2)	0 (0)		
Hemoglobin, g/L				0.686	Fisher
<115	105 (22)	72 (21)	33 (23)		
115–150	364 (76)	256 (76)	108 (76)		
>150	11 (2)	9 (3)	2 (1)		
Platelets, 10^9^/L				0.189	Fisher
<125	5 (1)	3 (1)	2 (1)		
125–350	398 (83)	274 (81)	124 (87)		
>350	77 (16)	60 (18)	17 (12)		
ALT, U/L				0.536	0.382
7–40	456 (95)	322 (96)	134 (94)		
>40	24 (5)	15 (4)	9 (6)		
AST, U/L				0.719	0.13
13–35	451 (94)	318 (94)	133 (93)		
>35	29 (6)	19 (6)	10 (7)		
Total protein, g/L				0.09	Fisher
<65	63 (13)	42 (12)	21 (15)		
65–85	415 (86)	295 (88)	120 (84)		
>85	2 (0)	0 (0)	2 (1)		
Albumin, g/L				1	0
<40	189 (39)	133 (39)	56 (39)		
40–55	291 (61)	204 (61)	87 (61)		
UREA, mmol/L				1	Fisher
<2.6	10 (2)	7 (2)	3 (2)		
2.6–7.5	458 (95)	321 (95)	137 (96)		
>7.5	12 (2)	9 (3)	3 (2)		
Creatinine, μmoI/L				0.898	0.215
<41	85 (18)	58 (17)	27 (19)		
41–73	377 (79)	266 (79)	111 (78)		
>73	18 (4)	13 (4)	5 (3)		
Uric acid, μmoI/L				0.66	0.83
<155	17 (4)	12 (4)	5 (3)		
155–357	432 (90)	301 (89)	131 (92)		
>357	31 (6)	24 (7)	7 (5)		
Glucose, mmol/L				0.34	Fisher
<3.9	3 (1)	2 (1)	1 (1)		
3.9–6.1	433 (90)	308 (91)	125 (87)		
>6.1	44 (9)	27 (8)	17 (12)		
Triglycerides, mmol/L				0.91	0.013
0.4–1.7	420 (88)	294 (87)	126 (88)		
>1.7	60 (12)	43 (13)	17 (12)		
Cholesterol, mmol/L				0.517	1.318
<3	17 (4)	13 (4)	4 (3)		
3–5.7	356 (74)	245 (73)	111 (78)		
>5.7	107 (22)	79 (23)	28 (20)		
HDL-C, mmol/L				0.532	0.39
>1.04	400 (83)	278 (82)	122 (85)		
≤1.04	80 (17)	59 (18)	21 (15)		
LDL-C, mmol/L				0.866	0.028
<3.6	399 (83)	279 (83)	120 (84)		
≥3.6	81 (17)	58 (17)	23 (16)		
GFR, mL/min				0.946	0.005
≤90	452 (94)	318 (94)	134 (94)		
>90	28 (6)	19 (6)	9 (6)		

Data are median (IQR) or n (%).

GS, grip strength; BMI, body mass index; PBF, Percentage Body Fat; VAS, visual analog scale; CCP, anti-cyclic citrullinated peptide antibody; RF, rheumatoid factor; CRP, C-reactive protein; ESR, erythrocyte sedimentation rate; DAS28-CRP, Disease Activity Score 28 with C-reactive protein; DAS28-ESR, Disease Activity Score 28 with the erythrocyte sedimentation rate; ALT, alanine aminotransferase; AST, aspartate aminotransferase; HDL-C, high-density lipoprotein; LDL-C, low-density lipoprotein; GFR, glomerular filtration rate.

### 3.2 Modeling of the nomogram prediction model

**Figure 2:**
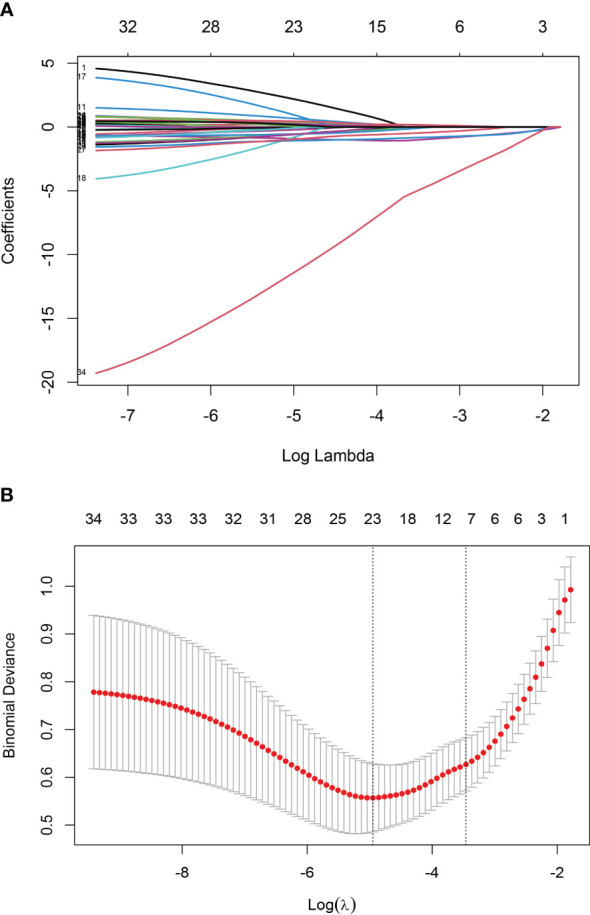
Screening variables using LASSO regression: **(A)** Path diagram illustrating LASSO regression coefficients; **(B)** Cross-validation curve for LASSO regression.

The training set underwent variable screening using LASSO regression analysis, with the path diagram of the regression coefficients and cross-validation curve shown in **Figure 2**. For optimal model fit, the λ corresponding to the time of the minimum mean square error (vertical dotted line on the left side of **Figure 2B**) was selected. LASSO regression analysis identified seven variables: body mass index (BMI), VAS, disease duration, hemoglobin, albumin, creatinine, and GS. These seven screening variables were included in the multifactor logistic regression analysis (**Table 2**), which revealed that BMI, disease duration, hemoglobin, and GS independently influenced RA sarcopenia, with a *P<0.05* for the regression coefficient significance test.

**Table 2:** Results of multifactorial logistic regression analysis of training sets complicating sarcopenia.

Variables	B	Wald	P-value	OR (95%Cl)
constant term (Intercept)	9.223	34.623	<0.001	–
BMI	-1.628	26.002	<0.001	0.196(0.1~0.353)
Disease duration	0.505	7.516	0.006	1.657(1.161~2.4)
hemoglobin	-1.544	15.757	<0.001	0.214(0.098~0.452)
GS	-0.289	22.958	<0.001	0.749(0.66~0.837)

GS, grip strength; BMI, body mass index; OR, odds ratio; CI, confidence interval.

**Figure 3:**
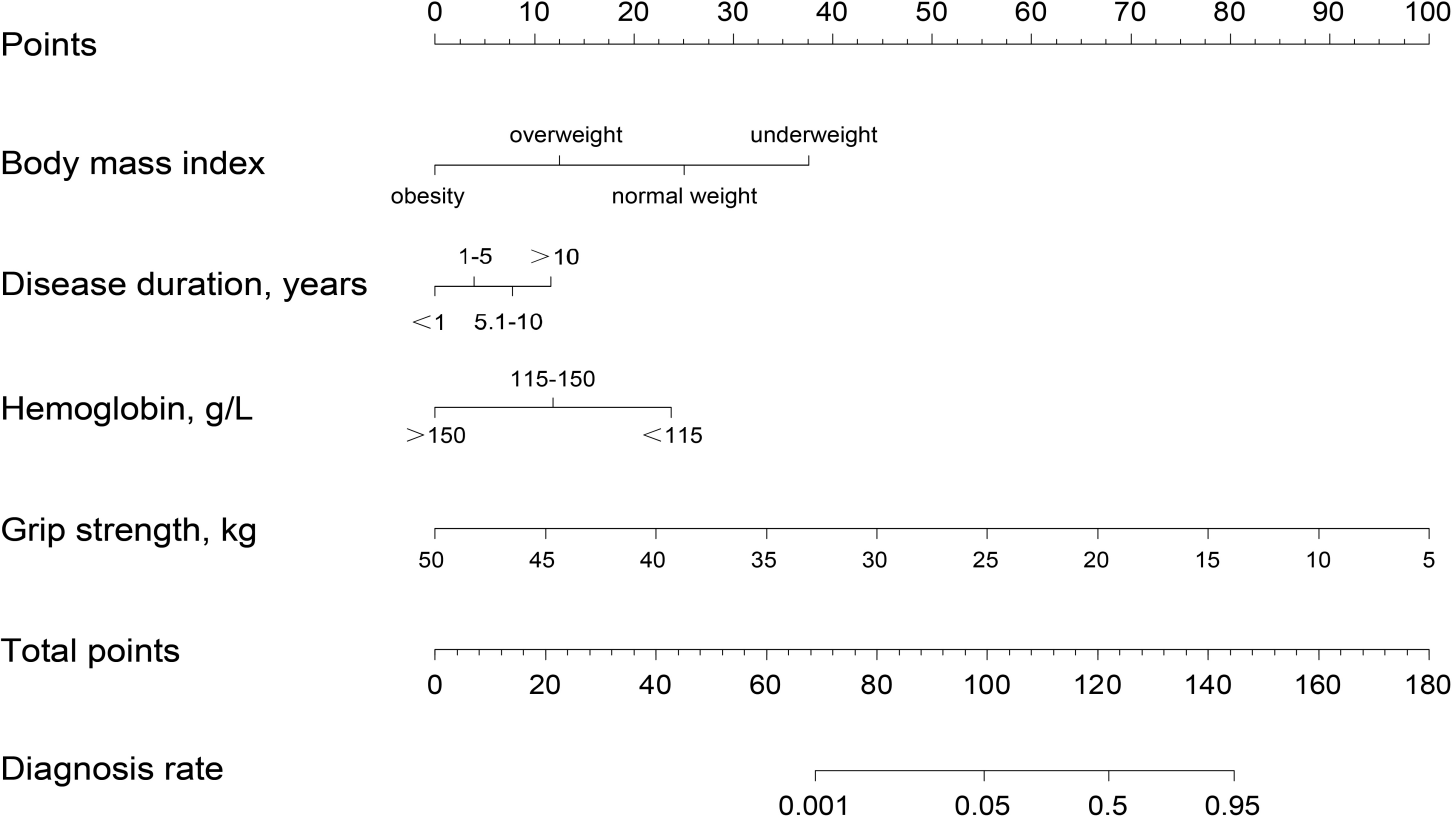
A nomogram prediction model for RA sarcopenia.

The study incorporated four independent risk factors—BMI, disease duration, hemoglobin, and GS—to develop a nomogram for predicting the risk of sarcopenia in patients with RA (**Figure 3**). The nomogram provides individual scores for each of the four independent risk factors, which are then summed to obtain a total score. The predicted probability corresponding to the total score indicates the likelihood of RA patients developing sarcopenia. The probability of sarcopenia in RA patients is approximately 5% when the total score on the nomogram reaches 100 and increases to 90% when the total score reaches 140.

### 3.3 Internal validation and evaluation of nomogram prediction models

**Figure 4:**
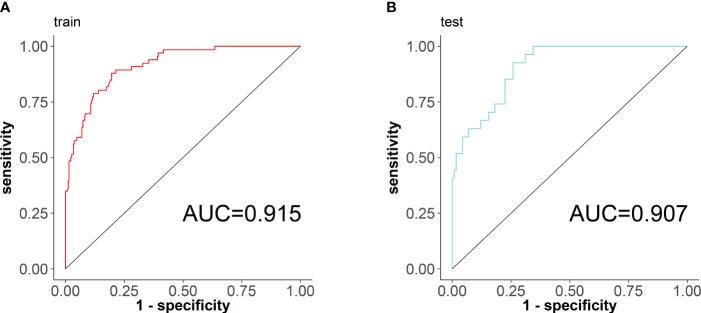
ROC curves of the nomogram prediction model for RA sarcopenia, with the x-axis denoting specificity and the y-axis denoting sensitivity. **(A)** ROC curve of the training set; **(B)** ROC curve of the validation set.

According to the results of the regression equation, the ROC curves were plotted using the data of the training set and the validation set respectively (**Figure 4**). The AUC of the training set is 0.915 (95% CI: 0.8795~0.9498), sensitivity 0.878, and specificity 0.804; the AUC of the validation set is 0.907 (95% CI: 0.8552~0.9597), sensitivity 0.925, specificity 0.741, indicating that the differentiation of the nomogram prediction model is good.

**Figure 5:**
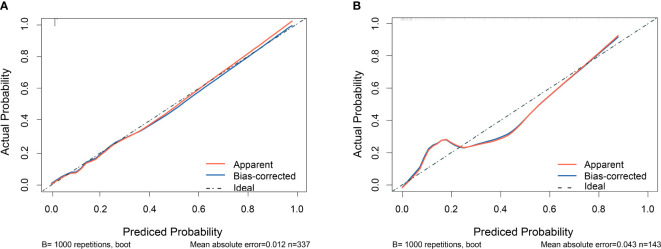
Calibration curves of the nomogram prediction model for RA sarcopenia. **(A)** calibration curve for the training set; **(B)** calibration curve for the validation set.

The calibration plots were drawn using data from the training set and the validation set, respectively (**Figure 5**). The horizontal axis represents the predicted probability of RA sarcopenia, while the vertical axis represents the actual percentage of patients with RA sarcopenia. The graph shows that the predicted values of the model are generally consistent with the actual values. The calibration ability of this prediction model was assessed using the Hosmer-Lemeshow goodness-of-fit test. The test results (*χ^2 ^= 6.6245, P = 0. 5776*) suggest that the difference between the predicted and actual values is not statistically significant (*P > 0.05*), indicating that the model is well-calibrated.

**Figure 6:**
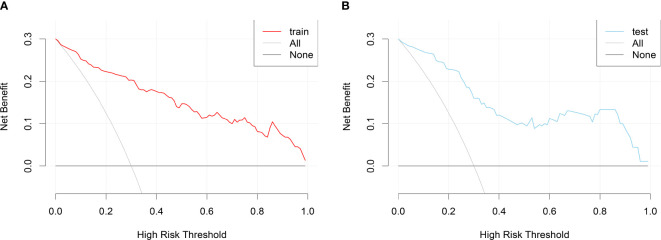
DCA curves for the nomogram prediction model of RA sarcopenia. **(A)** DCA curve for the training set; **(B)** DCA curve for the validation set.

DCA curves were plotted using the training set data and validation set data, respectively (**Figure 6**). DCA curves for both the training and validation sets demonstrated that patients could benefit from this novel prediction model across almost the entire range of 0% to 100%, suggesting that the prediction model has good clinical utility.

## 4 Discussion

To predict sarcopenia in RA patients, we developed a Nomogram model aimed at accurately estimating skeletal muscle mass and function using easily accessible examination findings. The predictive model demonstrated good discrimination and calibration, providing a net benefit across nearly the entire threshold range. To the best of our knowledge, this model is the first of its kind to accurately identify sarcopenia in RA patients.

The nomogram incorporates four clinically relevant variables: BMI, disease duration, hemoglobin, and GS. Data for these variables can be readily obtained from the medical records of RA patients. The GS instrument is inexpensive and portable, making the collection of GS data easy. Additionally, the model accounts for potential bias in GS data due to joint pain in RA patients, and combining GS with objective data, such as laboratory tests, improves the accuracy of predicting sarcopenia risk in this population. This is crucial as it enables clinicians to use routine data to accurately assess these risk factors and predict the risk of sarcopenia in RA patients.

The best way to diagnostically assess skeletal muscle mass is still undecided, and each has its advantages and disadvantages. The main assessment tools are computed tomography (CT), magnetic resonance imaging (MRI), dual-energy X-ray absorptiometry (DXA), BIA, muscle ultrasound, etc. (13). CT mainly scans at the third and fourth lumbar vertebral positions and utilizes muscle parameters to assess skeletal muscle mass, which has the disadvantages of higher cost and poor reproducibility of results (14). MRI is an extremely accurate test. MRI not only quantifies skeletal muscle, but also characterizes it, detecting fat deposits within skeletal muscle (15). However, MRI is very expensive, more used in scientific research, and not suitable for clinical promotion (16). DXA and BIA are two of the more commonly used methods, which do not contain ionizing radiation and are less costly. Primary care facilities are rarely equipped with this facility and can only be used in higher-level hospitals (17, 18). Muscle ultrasound can also be used to detect skeletal muscle mass, but it is less accurate and the results are more dependent on the level of the operator. Primary care providers may not have high-level technicians to detect skeletal muscle mass, limiting application scenarios (19). The prediction model constructed in this study has unique advantages, low cost, objective assessment data, reliable results, high repeatability, and high generalization value.

Nutritional variables like BMI and hemoglobin are frequently employed in clinical practice to evaluate the nutritional status of an individual, each possessing distinct characteristics. Studies have indicated that individuals with higher BMI are less likely to develop sarcopenia (20, 21). A high BMI is linked to a better prognosis and plays a significant role in reducing mortality, particularly in cardiovascular disease (22). Elevated BMI is linked to improved functional and cognitive status, particularly among men (23). However, in the RA population, the opposite is observed. Obesity can trigger low-grade inflammation in the body (24). RA patients with high BMI often show elevated disease activity and lower rates of achieving target-to-target (T2T) goals with treatment (25, 26). Weight loss could potentially improve the T2T rate in RA patients (27). Taking these studies into consideration, we should develop a more objective understanding of BMI. While a high BMI may reduce the risk of sarcopenia and lower the likelihood of falls and disability in RA patients, it does not align with contemporary RA treatment approaches. RA patients should focus on dietary supplementation to maintain a normal BMI, which can help reduce the risk of sarcopenia and align with standard treatment guidelines.

Our results indicate that hemoglobin is an independent risk factor for predicting RA sarcopenia. Hemoglobin is a key indicator for monitoring anemia and reflects the body’s nutritional status (28). Anemia, characterized by low hemoglobin levels, is a common hematologic disorder in patients (29), with a prevalence of about 72.8% in RA patients (30). Several factors can explain this, including iron deficiency, defective erythropoietin production, decreased bone marrow response to erythropoietin, and defective iron release from the reticuloendothelial system (31). Studies have shown that joint mobility is more severe in RA patients with anemia (32), interleukin-6 (IL-6), CRP, and CCP levels are significantly elevated in anemic RA patients compared to non-anemic RA patients, indicating high disease activity (31). Hemoglobin is a crucial predictor of sarcopenia. Studies on sarcopenia in older adults across various countries have demonstrated that low hemoglobin levels are linked to sarcopenia, muscle weakness, reduced physical function, fatigue, and respiratory distress (33, 34). Patients with higher hemoglobin levels exhibit faster gait speeds and stronger GS (28), reducing the risk of sarcopenia (35), consistent with our findings. Hemoglobin serves as a critical indicator of the inflammatory and nutritional status in RA patients, playing a pivotal role in predicting the development of RA sarcopenia. Early attention should be paid to hemoglobin levels in RA patients, with effective interventions. IL-6 receptor inhibitors and JAK inhibitors have been found to increase hemoglobin levels in RA patients with anemia (36, 37), potentially reducing the risk of RA sarcopenia with early use of these drugs.

GS is a key predictor of sarcopenia. In this study, GS was identified as an independent risk factor in the predictive risk nomogram. However, there was a discrepancy between the muscle strength results derived from the GS criteria and the skeletal muscle mass results in RA patients, which could be attributed to hand joint pain in these patients. Joint swelling and tenderness in RA patients have been shown to limit mobility and negatively affect muscle strength (8). GS meters are not fully accurate or objective in reflecting the true GS of RA patients during periods of moderate to high mobility. Studies should combine GS with objective indicators to improve the accuracy of predicting sarcopenia in RA patients. Hemoglobin levels have been found to correlate with muscle strength. Patients with low hemoglobin exhibited significantly weaker muscle strength compared to those with normal or higher hemoglobin levels (34). Similar findings were reported in studies related to RA, with BMI and disease duration also influencing muscle strength in RA patients (8). This study combined GS with disease duration, hemoglobin, and BMI—three objective and easily obtainable indicators—to significantly enhance the accuracy and objectivity of predicting sarcopenia risk in RA.

This study revealed that the duration of RA adversely affects both skeletal muscle mass and strength, with longer RA duration correlating with a higher risk of sarcopenia complications. The duration of RA is associated with weakened skeletal muscle strength (8). Additionally, the study revealed that patients with a longer duration of RA tend to have poorer nutritional status, which is associated with a 10% increased risk of developing sarcopenia each year (38). This conclusion was corroborated by a previous study conducted by Torii et al. (39). Utilizing RA duration, readily available information, as a key indicator in the predictive model of this study significantly reduces the data collection challenge and enables easy replication in all healthcare settings.

It is widely acknowledged that organismal inflammation is linked to muscle reduction in RA. Chronic inflammation disrupts the balance between muscle synthesis and atrophy, as evidenced by increased proteolysis and decreased muscle synthesis (8). The study revealed an association between sarcopenia and serum CRP levels as well as DAS28 scores, both of which are correlated with reduced skeletal muscle mass and strength (8, 40, 41). However, conflicting findings have also been reported. Barone et al. concluded that there was no association between inflammatory indicators and an increased risk of sarcopenia (42, 43). However, the present study found statistically significant differences in CRP, ESR, DAS28-CRP, and DAS28-ESR between the sarcopenia group and the non-sarcopenia group (**Supplementary Table 1** in the **Supplementary Material**), suggesting that inflammation may play a role in the development of sarcopenia. However, inflammation indicators did not emerge as independent risk factors and were not included in the nomogram. This may be attributed to the retrospective nature of this study, where inflammatory indicators can only reflect the inflammatory state at that stage, while sarcopenia develops over a prolonged period of pathology. Fibromyalgia and central nociceptive sensitization may also play a confounding role. In addition to the catabolic effects of organic inflammation on skeletal muscle, pain-induced limb wasting has an integral role in the development of sarcopenia (44, 45). Inflammation-induced high disease activity is traditionally thought to be the predominant mechanism of pain in RA, but there are still some patients who continue to have significant pain after inflammation is controlled, e.g., fibromyalgia, central nociceptive sensitization, and nociplastic pain (46, 47). The above conditions have the potential to contaminate disease activity indicators, resulting in sarcopenia being less associated with them. Prolonged disease activity monitoring may lead to new conclusions.

Except for sarcopenia, patients with RA are prone to other comorbidities. The correlation between common comorbidities of RA and sarcopenia has been studied. Interstitial lung disease (ILD) is a common complication of RA and is correlated with a marked increase in mortality in RA patients (48). Inflammatory disease-associated ILD was revealed to be a significant risk factor for declining muscle function, which may be attributed to the use of glucocorticoids in patients with ILD, hypoxemia leading to physical inactivity, and limb wasting (49). Osteoporosis is widespread among RA patients (50). Several studies have confirmed that sarcopenia and osteoporosis are strongly related, sarcopenia is a risk factor for osteoporosis (51). RA is known to be associated with an increased risk of tumors, which may be due to the pathophysiological characteristics of RA (52). Cancer-induced cachexia is an area of major importance in the field of sarcopenia. Cancer-induced anorexia, decreased activity, and a chronic inflammatory state of the body are critical factors in the development of sarcopenia (4). Patients with RA combined with tumors have a substantial increase in the probability of complications of sarcopenia (4). RA patients have a complex condition, and according to the above study results, RA comorbidities have a detrimental effect on the progression of sarcopenia. Clinical attention should be focused on all types of comorbidities along with the treatment of the RA disease itself.

The use of RA therapeutic agents [e.g., glucocorticoids, nonsteroidal anti-inflammatory drugs (NSAIDs), conventional synthetic disease-modifying antirheumatic drugs (csDMARDs), and biologically/targeted synthetic disease-modifying antirheumatic drugs (b/tsDMARDs)] may have a role in sarcopenia formation, but this has not been definitively determined. NSAIDs are commonly used in RA patients. A study finds NSAIDs reduce the risk of sarcopenia in older patients by 80% (53). However, another study on cancer cachexia showed the opposite result. The evidence for recommending NSAIDs for the treatment of patients with cancer cachexia is insufficient (54). Studies have shown that glucocorticoids are strongly associated with sarcopenia (55, 56), that the use of csDMARDs is negatively associated with sarcopenia (55), and that b/tsDMARDs do not show a correlation with sarcopenia formation (55, 57). Some studies present inconsistent conclusions that NSAIDs, bDMARDs, and glucocorticoids are all risk factors for sarcopenia (39, 58). Currently, on the basis of standardized treatment, both clinicians and patients are more attentive to the maintenance and improvement of quality of life. The correlation between drugs and sarcopenia still requires the emphasis of researchers, which may be a new direction for the selection of therapeutic drugs for RA in the future.

Sarcopenia is an important risk factor for falls and fractures in RA patients. Multiple studies support this conclusion (6, 59, 60). Sarcopenia elevates the risk of vertebral osteoporotic fractures (VOFP) in RA patients. The probability of VOFP for RA sarcopenia was elevated by 10% compared to patients with uncomplicated sarcopenia (6). High skeletal muscle mass is protective against VOPF (59). This may be related to the fact that people with sarcopenia have a higher degree of joint damage and poorer joint function and body balance, leading to an increased chance of falling (60). Due to the retrospective nature of this study, it was not possible to know the subsequent falls and fractures of the patients. Future prospective studies will be conducted in the hope of obtaining more clinically valuable conclusions. Enhancing skeletal muscle mass and strength and delaying the onset of RA sarcopenia is very meaningful for the quality of life of RA patients.

Screening and timely intervention for sarcopenia associated with RA is critical. Studies have found that supplementation with nutrients such as folic acid and vitamin D (61), adherence to scientifically based dietary support, and regular physical activity can enhance muscle mass and strength and provide specific health benefits for people with RA (8, 62, 63). As many as 66% of RA patients have been reported to have inadequate dietary nutrient intake and may suffer from malnutrition, contributing to an increased risk of sarcopenia (38). However, the nutritional status of RA patients goes unnoticed by clinicians (64). We should be aware of the tremendous benefits of dietary nutritional support for RA improvement and quality of life. Clinicians should individualize nutritional prescriptions for patients while treating RA. Resistance exercise is recommended as a first-line treatment for sarcopenia (62, 65). Patients with RA are often accompanied by swollen and painful joints, and blind resistance exercise may have little effect. Clinicians should carefully assess the physical condition of patients with RA, and individualized resistance training when the disease is low activity and the organism is pain-free can slow the progression of sarcopenia and maintain organismal function. Nutrient deficiencies such as vitamin D, vitamin B12, and folic acid have been found to have a detrimental effect on muscle function (61, 66). Folic acid is essential for the development and function of skeletal muscle (67), and folate levels are significantly correlated with both muscle mass and strength (68, 69). This evidence suggests that adequate dietary and nutrient supplementation support and appropriate resistance training are important components of a strategy to treat RA sarcopenia. Clinical application of this study’s model for risk prediction of sarcopenia in RA patients and early therapeutic interventions in high-risk patients can be of great benefit to the quality of patient survival.

Notably, the primary objective of this study was to develop straightforward and practical predictive tools for identifying sarcopenia in RA patients. The four indicators (BMI, hemoglobin, GS, and disease duration) included in the current study were well-calibrated and differentiated, confirming the model’s good performance. The DCA curve indicated that the present model showed benefits across almost the full threshold range, aiding clinicians in improving their ability to screen for RA sarcopenia, assess patient prognosis, and make early decisions, with good clinical promotion value.

Our study has several limitations. Firstly, This study is retrospective and may be prone to bias. For instance, tracking the progression of disease activity in RA patients over time was not feasible, highlighting the need for prospective studies to clarify if this is a contributing factor. However, this limitation did not affect the construction of the predictive model for the risk of RA sarcopenia. Secondly, the study was conducted at a single center. Although preliminary validation of the risk scoring model’s performance was done with an independent cohort, evaluation at multiple medical centers is necessary to ensure its broad applicability and reliability. Thirdly, despite the substantial inclusion of Chinese patients in the study, future studies should expand to Asia or other geographic regions, considering that geographic and ethnic differences may affect disease performance and risk scores. For future study planning, we propose to test this risk score model in different clinical settings and multiple national contexts to fully validate its accuracy in predicting risk.

## 5 Conclusion

In conclusion, this study constructed and validated a risk prediction model for RA sarcopenia to identify RA patients at risk of sarcopenia early. The variables in the model are easily accessible and can help clinicians at all levels to improve RA sarcopenia screening, intervene promptly and effectively, and enhance the prognosis and quality of life of RA patients.

## Data availability statement

The raw data supporting the conclusions of this article will be made available by the authors, without undue reservation.

## Ethics statement

The studies involving humans were approved by Ethics Committee of Shandong University of Traditional Chinese Medicine Affiliated Hospital. The studies were conducted in accordance with the local legislation and institutional requirements. The ethics committee/institutional review board waived the requirement of written informed consent for participation from the participants or the participants’ legal guardians/next of kin because This was a retrospective study and did not involve patients’ private information.

## Author contributions

YQ: Writing – original draft, Writing – review & editing, Data curation, Investigation, Methodology, Project administration, Software, Validation. LZ: Data curation, Project administration, Writing – original draft. YL: Data curation, Formal analysis, Writing – original draft. YF: Formal analysis, Writing – original draft. MW: Formal analysis, Project administration, Writing – original draft. CL: Formal analysis, Methodology, Writing – review & editing. XW: Investigation, Project administration, Writing – original draft. YW: Investigation, Project administration, Writing – review & editing. BX: Data curation, Formal analysis, Writing – review & editing. QZ: Data curation, Supervision, Writing – review & editing. YCL: Project administration, Validation, Writing – review & editing. PJ: Methodology, Project administration, Writing – review & editing, Supervision.
