# Supplementary material for: Development and validation of a predictive model assessing the risk of sarcopenia in rheumatoid arthritis patients
Source: Front Immunol. 2024 Jul 29;15:1437980. doi: 10.3389/fimmu.2024.1437980 (PMC11317408; doi:10.3389/fimmu.2024.1437980)
Supplement: Supplementary file 1 [file Table_1.docx]

**Table 1 Comparison of clinical data between sarcopenia and non-sarcopenia in RA patients.**

| Variables | Total  (n = 337) | Non-Sarcopenia  (n = 271) | Sarcopenia  (n = 66) | P-value | χ^2^/Fisher |
| --- | --- | --- | --- | --- | --- |
| Sex |  |  |  | 0.491 | 0.475 |
| Female | 301 (89) | 240 (89) | 61 (92) |  |  |
| Male | 36 (11) | 31 (11) | 5 (8) |  |  |
| Age, years |  |  |  | 0.018 | 8.023 |
| ＜44 | 87 (26) | 72 (27) | 15 (23) |  |  |
| 45-59 | 176 (52) | 148 (55) | 28 (42) |  |  |
| 60-74 | 74 (22) | 51 (19) | 23 (35) |  |  |
| Disease duration, years |  |  |  | < 0.001 | 35.344 |
| ＜1 | 120 (36) | 108 (40) | 12 (18) |  |  |
| 1-5 | 124 (37) | 106 (39) | 18 (27) |  |  |
| 5.1-10 | 52 (15) | 36 (13) | 16 (24) |  |  |
| ＞10 | 41 (12) | 21 (8) | 20 (30) |  |  |
| GS, kg | 19.35 (16.5, 24.15) | 20.95 (17.52, 25.55) | 15.52(13.81, 17.51) | < 0.001 | 14973.5 |
| BMI |  |  |  | < 0.001 | Fisher |
| underweight | 13 (4) | 2 (1) | 11 (17) |  |  |
| normal weight | 127 (38) | 84 (31) | 43 (65) |  |  |
| overweight | 132 (39) | 122 (45) | 10 (15) |  |  |
| obesity | 65 (19) | 63 (23) | 2 (3) |  |  |
| PBF |  |  |  | < 0.001 | 40.477 |
| normal | 47 (14) | 24 (9) | 23 (35) |  |  |
| mildly obese | 112 (33) | 85 (31) | 27 (41) |  |  |
| moderately obese | 88 (26) | 78 (29) | 10 (15) |  |  |
| severe obesity | 90 (27) | 84 (31) | 6 (9) |  |  |
| VAS |  |  |  | < 0.001 | Fisher |
| pain-free | 106 (31) | 101 (37) | 5 (8) |  |  |
| Mild pain | 148 (44) | 116 (43) | 32 (48) |  |  |
| Moderate pain | 76 (23) | 50 (18) | 26 (39) |  |  |
| Severe pain | 7 (2) | 4 (1) | 3 (5) |  |  |
| CCP, RU/ml |  |  |  | 0.54 | Fisher |
| ＜25 | 33 (10) | 24 (9) | 9 (14) |  |  |
| 25-75 | 16 (5) | 13 (5) | 3 (5) |  |  |
| ＞75 | 288 (85) | 234 (86) | 54 (82) |  |  |
| RF, IU/ml |  |  |  | 0.416 | 1.753 |
| ＜15 | 40 (12) | 35 (13) | 5 (8) |  |  |
| 15-45 | 61 (18) | 50 (18) | 11 (17) |  |  |
| ＞45 | 236 (70) | 186 (69) | 50 (76) |  |  |
| CRP, mg/L |  |  |  | 0.032 | 6.901 |
| ＜3.12 | 161 (48) | 139 (51) | 22 (33) |  |  |
| 3.12-10 | 58 (17) | 44 (16) | 14 (21) |  |  |
| ＞10 | 118 (35) | 88 (32) | 30 (45) |  |  |
| ESR, mm/hr |  |  |  | 0.004 | 10.817 |
| ＜20 | 141 (42) | 120 (44) | 21 (32) |  |  |
| 20-60 | 157 (47) | 127 (47) | 30 (45) |  |  |
| ＞60 | 39 (12) | 24 (9) | 15 (23) |  |  |
| DAS28-ESR |  |  |  | 0.047 | 7.964 |
| Relief | 112 (33) | 99 (37) | 13 (20) |  |  |
| Low activity | 36 (11) | 29 (11) | 7 (11) |  |  |
| Medium Activity | 159 (47) | 122 (45) | 37 (56) |  |  |
| High activity | 30 (9) | 21 (8) | 9 (14) |  |  |
| DAS28-CRP |  |  |  | < 0.001 | Fisher |
| Relief | 133 (39) | 118 (44) | 15 (23) |  |  |
| Low activity | 81 (24) | 68 (25) | 13 (20) |  |  |
| Medium Activity | 108 (32) | 73 (27) | 35 (53) |  |  |
| High activity | 15 (4) | 12 (4) | 3 (5) |  |  |
| White blood cells, 10^9^/L |  |  |  | 0.016 | Fisher |
| ＜3.5 | 16 (5) | 11 (4) | 5 (8) |  |  |
| 3.5-9.5 | 305 (91) | 251 (93) | 54 (82) |  |  |
| ＞9.5 | 16 (5) | 9 (3) | 7 (11) |  |  |
| Neutrophil, 10^9^/L |  |  |  | 0.058 | Fisher |
| ＜1.8 | 16 (5) | 10 (4) | 6 (9) |  |  |
| 1.8-6.3 | 306 (91) | 251 (93) | 55 (83) |  |  |
| ＞6.3 | 15 (4) | 10 (4) | 5 (8) |  |  |
| Lymphocyte, 10^9^/L |  |  |  | 0.35 | Fisher |
| ＜1.1 | 42 (12) | 36 (13) | 6 (9) |  |  |
| 1.1-3.2 | 286 (85) | 229 (85) | 57 (86) |  |  |
| ＞3.2 | 9 (3) | 6 (2) | 3 (5) |  |  |
| Neutrophil % |  |  |  | 0.123 | Fisher |
| ＜40 | 6 (2) | 3 (1) | 3 (5) |  |  |
| 40-70 | 276 (82) | 225 (83) | 51 (77) |  |  |
| ＞70 | 55 (16) | 43 (16) | 12 (18) |  |  |
| Lymphocyte % |  |  |  | 0.424 | Fisher |
| ＜20 | 56 (17) | 46 (17) | 10 (15) |  |  |
| 20-50 | 276 (82) | 222 (82) | 54 (82) |  |  |
| ＞50 | 5 (1) | 3 (1) | 2 (3) |  |  |
| Red blood cells, 10^12^/L |  |  |  | < 0.001 | Fisher |
| ＜3.8 | 62 (18) | 36 (13) | 26 (39) |  |  |
| 3.8-5.1 | 267 (79) | 228 (84) | 39 (59) |  |  |
| ＞5.1 | 8 (2) | 7 (3) | 1 (2) |  |  |
| Hemoglobin, g/L |  |  |  | < 0.001 | Fisher |
| ＜115 | 72 (21) | 34 (13) | 38 (58) |  |  |
| 115-150 | 256 (76) | 230 (85) | 26 (39) |  |  |
| ＞150 | 9 (3) | 7 (3) | 2 (3) |  |  |
| Platelets, 10^9^/L |  |  |  | 0.178 | Fisher |
| ＜125 | 3 (1) | 3 (1) | 0 (0) |  |  |
| 125-350 | 274 (81) | 225 (83) | 49 (74) |  |  |
| ＞350 | 60 (18) | 43 (16) | 17 (26) |  |  |
| ALT, U/L |  |  |  | 1 | Fisher |
| 7-40 | 322 (96) | 259 (96) | 63 (95) |  |  |
| ＞40 | 15 (4) | 12 (4) | 3 (5) |  |  |
| AST, U/L |  |  |  | 0.773 | Fisher |
| 13-35 | 318 (94) | 256 (94) | 62 (94) |  |  |
| ＞35 | 19 (6) | 15 (6) | 4 (6) |  |  |
| Total protein, g/L |  |  |  | < 0.001 | 11.825 |
| ＜65 | 42 (12) | 25 (9) | 17 (26) |  |  |
| 65-85 | 295 (88) | 246 (91) | 49 (74) |  |  |
| ＞85 |  |  |  | < 0.001 | 26.854 |
| Albumin, g/L | 133 (39) | 88 (32) | 45 (68) |  |  |
| ＜40 | 204 (61) | 183 (68) | 21 (32) |  |  |
| 40-55 |  |  |  | 0.603 | Fisher |
| Urea, mmol/L | 7 (2) | 7 (3) | 0 (0) |  |  |
| ＜2.6 | 321 (95) | 257 (95) | 64 (97) |  |  |
| 2.6-7.5 | 9 (3) | 7 (3) | 2 (3) |  |  |
| ＞7.5 |  |  |  | 0.004 | Fisher |
| Creatinine, μmoI/L | 58 (17) | 37 (14) | 21 (32) |  |  |
| ＜41 | 266 (79) | 223 (82) | 43 (65) |  |  |
| 41-73 | 13 (4) | 11 (4) | 2 (3) |  |  |
| ＞73 |  |  |  | 0.12 | Fisher |
| Uric acid, μmoI/L | 12 (4) | 7 (3) | 5 (8) |  |  |
| ＜155 | 301 (89) | 243 (90) | 58 (88) |  |  |
| 155-357 | 24 (7) | 21 (8) | 3 (5) |  |  |
| ＞357 |  |  |  | 0.486 | Fisher |
| Glucose, mmol/L | 2 (1) | 1 (0) | 1 (2) |  |  |
| ＜3.9 | 308 (91) | 248 (92) | 60 (91) |  |  |
| 3.9-6.1 | 27 (8) | 22 (8) | 5 (8) |  |  |
| ＞6.1 |  |  |  | 1 | 0 |
| Triglycerides, mmol/L | 294 (87) | 236 (87) | 58 (88) |  |  |
| 0.4-1.7 | 43 (13) | 35 (13) | 8 (12) |  |  |
| ＞1.7 |  |  |  | 0.93 | Fisher |
| Cholesterol, mmol/L | 13 (4) | 10 (4) | 3 (5) |  |  |
| ＜3 | 245 (73) | 197 (73) | 48 (73) |  |  |
| 3-5.7 | 79 (23) | 64 (24) | 15 (23) |  |  |
| ＞5.7 |  |  |  | 0.984 | 0 |
| HDL-C, mmol/L | 278 (82) | 223 (82) | 55 (83) |  |  |
| ＞1.04 | 59 (18) | 48 (18) | 11 (17) |  |  |
| ≤1.04 |  |  |  | 0.755 | 0.098 |
| LDL-C, mmol/L | 279 (83) | 223 (82) | 56 (85) |  |  |
| ＜3.6 | 58 (17) | 48 (18) | 10 (15) |  |  |
| ≥3.6 |  |  |  | 0.55 | Fisher |
| GFR, mL/min | 318 (94) | 257 (95) | 61 (92) |  |  |
| ≤90 | 19 (6) | 14 (5) | 5 (8) |  |  |
| ＞90 |  |  |  |  |  |

*Note: Data are median (IQR) or n (%).*

Abbreviations: GS, grip strength; BMI, body mass index; PBF, Percentage Body Fat; VAS, visual analog scale; CCP, anti-cyclic citrullinated peptide antibody; RF, rheumatoid factor; CRP, C-reactive protein; ESR, erythrocyte sedimentation rate; DAS28-CRP, Disease Activity Score 28 with C-reactive protein; DAS28-ESR, Disease Activity Score 28 with the erythrocyte sedimentation rate; ALT, alanine aminotransferase; AST, aspartate aminotransferase; HDL-C, high-density lipoprotein; LDL-C, low-density lipoprotein; GFR, glomerular filtration rate.
